# Odds of Anxiety and Depression Symptoms in School-Aged Children From Official Language Minority Communities

**DOI:** 10.3389/fpubh.2021.660041

**Published:** 2021-06-23

**Authors:** Jérémie B. Dupuis, Jimmy Bourque, Salah-Eddine El Adlouni

**Affiliations:** ^1^Faculté des Sciences de l'éducation, Université de Moncton, Moncton, NB, Canada; ^2^Royal College of Physicians and Surgeons of Canada, Ottawa, ON, Canada; ^3^Faculté des Sciences, Département de mathématiques et de Statistique, Université de Moncton, Moncton, NB, Canada

**Keywords:** public health, epidemiology, determinants of health, mental health, OLMC

## Abstract

**Objectives:** The aim of this paper is to assess the odds of suffering from anxiety or depression symptoms based on the presence of certain determinants of health for youth living in the province of New Brunswick, Canada, and in two linguistically different Official Language Minority Communities (OLMCs) in the same province.

**Methods:** With a sample of 22,329 students from grades 7 to 12 in the province of New Brunswick, Canada, logistic regressions were performed to assess each determinant of health's effect on symptoms of anxiety and depression.

**Results:** Some social determinants, like family support, social support and food insecurity, were identified as important determinants of mental health status regardless of linguistic group membership or community membership, while other determinants, such as alcohol use, cannabis use and natural environment, were more prominent in one OLMC than the other.

**Discussion:** Social psychology and public health theories are used in an attempt to explain the results. Limitations and recommendations are also brought forward.

## Introduction

Recent data on New Brunswick grade school students suggest mental health challenges. The New Brunswick Health Council's Student Wellness Survey (NBSWS) assessed the health, wellness attitudes and behaviors of 32,677 students from grade 7 to 12 (1). The survey revealed that only two out of three students (65.7%) consider themselves in good or excellent general health. As for mental health, the NBSWS data revealed that 33.5% of students between grades 7 and 12 felt nervous, anxious or on edge every day for at least 2 weeks in the previous 12 months and 31.7% felt sad or hopeless every day for at least 2 weeks in the previous 12 months. Although symptoms in early adolescence don't always lead to a diagnosed anxiety or depression disorder, it has been reported that 10.4 and 11.5% of New Brunswick's population between the ages of 15 and 25 suffer from a Depressive Disorder or a Generalized Anxiety Disorder, respectively (2). The aim of this paper is to assess the odds of suffering from anxiety or depression symptoms based on the presence of certain determinants of health for youth living in two linguistically different Official Language Minority Communities (OLMCs) in New Brunswick and for the province as a whole.

**Figure 1:**
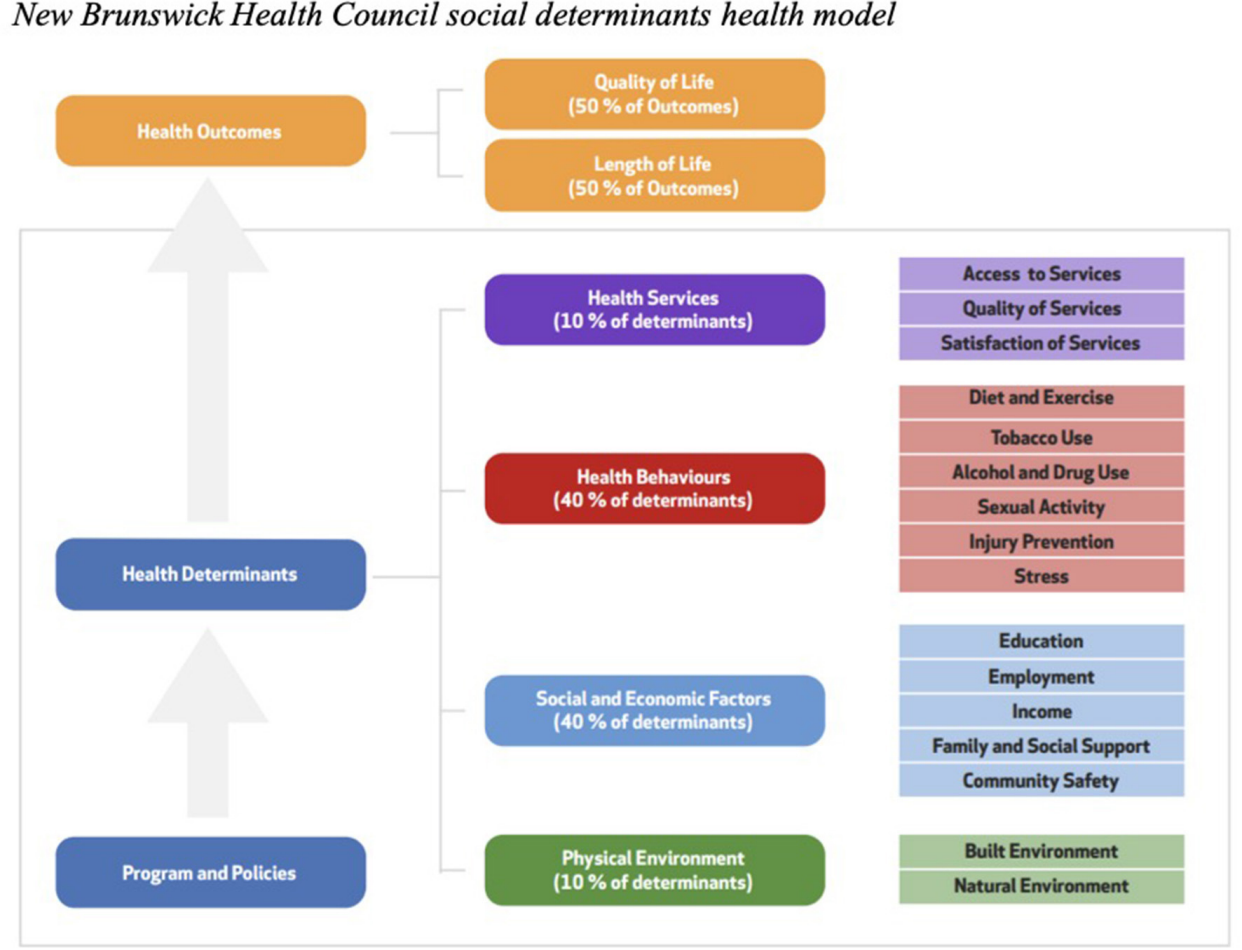
New Brunswick health council social determinants health model.

To address such issues, the New Brunswick Health Council (NBHC) adopted a province-specific epidemiological model, the Social Determinants of Health model, in 2014 (Figure 1). In this model, health services, health behaviors, social and economic factors and physical environment features are all included as health determinants, meaning that individual and population-level factors are considered. These health determinants directly affect selected health outcomes. For this study, the emphasis was put on mental health as the outcome. The goal of this study is to assess the odds of students having symptoms of anxiety and depression based on the presence or absence of social determinants of health.

### Indicators and Empirical Evidence Related to Mental Health

Mental health is not simply an absence of mental disorder. It is “a state of well-being in which every individual realizes his or her own potential, can cope with the normal stresses of life, can work productively and fruitfully, and is able to make a contribution to her or his community” (3). In the following paragraph, we will review the literature to identify determinants linked to youth mental health.

#### Diet and Food Insecurity

Melchior et al. (4) used data from the Longitudinal Study of Child Development in Quebec to determine the effect of food insecurity on children's mental health. From their total sample of 2,120 children, the prevalence of food insecurity was just 5.6%, but from this sample the authors concluded that children living with food insecurity were 1.79 (1.15, 2.79) times more likely to experience symptoms of anxiety and depression. In New Brunswick, a recent study suggested that 21% of children suffers from food insecurity due to family financial constraints (5), a percentage that is higher than Quebec's 16% or Ontario's 17%, but close to the other two maritime provinces.

#### Tobacco, Alcohol and Cannabis Use

Smoking and mental health are intimately linked. In Canada, 13% of the population are considered regular smokers. When Canadians were asked to self-evaluate their mental health based on depression, anxiety, and mood disorders, 77.4% of non-smokers rated themselves as having excellent or very good mental health, while only 65.0% of current smokers said the same (6). Fergusson et al. (7) tested the causal links between alcohol abuse or dependence and major depression. Controlling for other variables such as life stress, cannabis use, other illicit drug use, affiliation with deviant peers, unemployment, partner substance use, and partner criminality, findings suggest that the associations between alcohol abuse or dependence and major depression were best explained by a causal model in which problems with alcohol led to increased risk of major depression, as opposed to a self-medication model in which major depression led to increased risk of alcohol abuse or dependence.

As for drug use and mental health (8), analyzed data from the 2002 Canadian Community Health Survey (*n* = 36,984) and reported that illicit substance users were 2.45 (1.89, 3.16) times more likely than the general population to have mood disorders (depression and/or mania) and 2.30 (1.75, 3.03) times more likely to have an anxiety disorder. When substance dependence was present, these figures jumped to 5.74 (4.07, 8.08) and 4.76 (3.28, 6.91) times for mood and anxiety disorders, respectively. More recently (9), explored the association between cannabis and depression in adolescents. With four cohorts and a total of 6,900 participants, increased use of cannabis led to increased symptoms of depression. When confounding variables were analyzed, the strength of this association was reduced, but was still significant. However, the direction of the causality could not be established with the available data.

#### Sexual Activity

The effects of sexual activity on mental health are a topic rarely covered in science, especially for children and adolescents. Most studies on adolescents tend to look at the effects of sexual orientation and sexual identity on mental health, rather than sexual activity. For example (10), studied the effects of sexual orientation for Canadian adolescents. Results from these studies indicate that 29% of bisexual male teens and 35% of gay teens report feeling emotional distress, which includes being sad, discouraged or hopeless, in the last month. These numbers are much higher than their heterosexual male colleagues, with only 13% of them reporting the same. For adolescent girls, 50% of bisexual teens and 61% of lesbian teens report emotional distress, vs. only 22% of their heterosexual female colleagues. The above-mentioned study illustrates that sexual orientation and identity is an important predictor of mental health issues, adding to the importance of considering it as a social determinant of health. However, it's important to note that being lesbian, gay, bisexual or transgender is not the cause of mental health problems. In fact, lack of social or family support may be the root cause of these issues whereas social support gained from joining other members of the LGBTQ+ community may serve as a protective factor.

#### Employment and Income

Socioeconomic status (SES) is one of the most influential social determinants of mental health for children and adolescents. For the Canadian context (11), conducted a literature review of the effects of SES on mental health. They concluded that for Canadian youth between the ages of 10 and 15, SES had a negative association with the prevalence of depressed mood and anxiety. Higher rates of depressed mood and anxiety among low SES youth may impact emotional development and limit future educational and occupational achievement, which in turn limits the probability of increasing SES in the future. Similar conclusions were reached by (12). According to the American Psychological Association (13), SES is directly related to family resources, youth career aspirations, and youth psychological well-being. Unfortunately, low educational achievement limits the individual's ability to shed the effects associated with low socioeconomic status, thus becoming a generationally recurring problem (13).

#### Family and Social Support

Family and social support are described as the perceived regard and support that significant others, such as friends and family members, grant the individual (14). In their study with 273 Canadian 7th grade students (14), found that classmates' social support reduced internalized behaviors, such as anxiety, depression and withdrawal, while parental support played a key role in reducing externalized behaviors, such as aggression and behavior problems. Other studies support these findings, suggesting a well-established link between available social support and mental health in adolescents (15), and for LGBT youth, where lack of social support translated into higher levels of psychological distress (16).

In their multilevel investigation of the effects of social support on adolescent mental health (17), found that perceived sub-optimal social support was associated with high symptomology of depression. Interestingly, SES was also linked to perceived social support, meaning that low SES adolescents had lower perceived social support. Finally (18), conducted a longitudinal study with 2,616 students from 23 different Canadian high schools. They studied the effects of social support from classmates, teachers and family on mental health problems. Results showed that even when controlling for family social support, student perceptions of declining social support from friends and teachers each contributed to predicting decreasing self-esteem and increasing depression. Low social support from friends was the only predictor of social anxiety.

#### Physical Environment and Mental Health

The built environment is important for both mental and physical health and can often reduce the risks associated with communicable diseases, mental disorders, and poor mental health (19). In fact, interventions that address community determinants, like improving people's sense of social belonging, strengthening community networks, building social capital, improving neighborhood environments and community safety, tend to support mental health (20). Similarly, interventions that target sanitation, water supply, infrastructure upgrades, transportation, environmental hazards, waste management and housing often lead to improvement in physical and mental health (21). Living close to natural environments that allow outdoor activities such as walking, running, cycling, and gardening have known benefits for mental health issues like anxiety and depression (22).

### Minority Context

New Brunswick is recognized by the *Canadian Charter of Rights and Freedoms* as having French and English as its two official languages and is Canada's only officially bilingual province. The Charter also mentions that Francophone and Anglophone communities share the same rights, privileges and status. Despite 68.2% of the province's population indicating English as the language most spoken at home, French remains widespread in some areas, such as the Madawaska region, where 88.4% of citizens declare French as the language spoken at home (23). In other regions, Fredericton/River Valley for example, only 3.0% of the population use French at home (23). Inevitably, the overwhelming majority of social interactions in communities like Fredericton will be in English, resulting in greater chances of assimilation for the Francophone community (24, 25). However, some authors have suggested that Francophones in Francophone majority communities could possibly be isolated from the assimilation forces common to most Francophones in the province, as their local strength in numbers could shield them from the mental health pitfalls often associated with minority group membership (26).

Research on the mental health status of official language minority communities (OLMCs) yields mixed results. On the one hand, the prevalence of mental health problems among Francophones living in a minority context is higher than among non-Francophones (27, 28). On the other hand, Francophones and Anglophones living in minority settings do not always experience a higher rate of mental health problems (29, 30). In New Brunswick, Francophones seem to have lower rates of mental health issues than their Anglophone counterparts, regardless of the linguistic setting. In fact, fewer Francophone students have symptoms of depression (27.8%) than Anglophone students (36.1%) in the Anglophone majority community of Fredericton and in the Francophone majority region of Madawaska (22.3% for Francophones vs. 36.6% for Anglophones). Although less divergent, this tendency is also present for symptoms of anxiety. These statistics reveal a discrepancy between the typical OLMCs literature and what is observed in New Brunswick, highlighting the need for further research.

As such, the goal of this study is to assess the odds of anxiety and depression symptoms in OLMCs in New Brunswick by establishing which social determinants of health are associated with these symptoms. As this research compares school-aged children from two linguistically different OLMCs, it is hypothesized that social determinants that are close to the individual, such as family, friends and the school environment, will play an important role on their mental health status.

## Methods

### Study Sample

The NBHC's New Brunswick Student Wellness Survey (NBSWS) is a cross-sectional survey that aims to “examine students' perceptions, attitudes and behaviors in a number of key areas related to student wellness” (NBHC, paragraph 1, 2018), and is done in cooperation with the Department of Education and Early Childhood Development. This survey was completed in May 2016. All schools in the province were invited to participate. In total, data from 32,677 students was gathered, representing 69.9% of the total student population (1). After preliminary analyses were done on the dataset, 22,329 students remained. Of the remaining 22,329 students, 4,490 are from the Anglophone majority region of Fredericton and 1,580 are from the Francophone majority region of Madawaska, the regions chosen to represent Francophone and Anglophone OLMCs, respectively.

As for demographic variables, gender, spoken language, indigenous status, immigration status, special needs status, and LGBTQ+ were considered, as they have been associated with mental health (31–33) or are often used by the NBHC (1). For the most part, language was inferred from the language of the school the questionnaire was filled in. The exception to this rule being for French immersion students, in other words, Anglophone students who are learning French by having the majority of their school curriculum in French, who were given English questionnaires in order to avoid biases in comprehension. These students were considered to be Anglophone students by the survey. Participants who respond positively to indigenous status, immigration status, special needs status and/or LGBTQ+ were attributed a score of 1, and 0 otherwise. All demographic variables were taken from the NBSWS. See Table 1 for descriptive statistics of the study sample.

**Table 1:** Descriptive statistics of samples.

	**New Brunswick** ***N* = 22,329**	**Fredericton** **(French OLMC)** ***N* = 4,490**	**Madawaska** **(English OLMC)** ***N* = 1,580**
Francophones	6,599 (29.6%)	415 (9.2%)	1,379 (87.3%)
Anglophones	15,730 (29.6%)	4,075 (90.8%)	201 (12.7%)
Males	10,255 (45.9%)	2,100 (46.8%)	681 (43.1%)
Females	11,792 (52.8%)	2,323 (51.7%)	886 (56.1%)
Independent gender	282 (1.3%)	67 (1.5%)	13 (0.8%)
Average age	14.7	14.7	14.8
LGBTQ+	3,129 (14.0%)	712 (15.9%)	214 (13.5%)
Aboriginal status	1,172 (5.2%)	322 (7.2%)	49 (3.1%)
Immigrant status	1,130 (5.1%)	313 (7.0%)	36 (2.3%)
Special learning needs	3,856 (17.3%)	749 (16.7%)	209 (13.2%)

### Outcome Variables

The outcome variables were taken from the NBSWS. Symptoms of depression were measured with “*During the past 12 months, did you ever feel so sad or hopeless almost every day for 2 weeks or more in a row that you stopped doing some usual activities?*,” while symptoms of anxiety were measured with “*During the past 12 months, did you ever feel nervous, anxious or on edge or were you unable to stop or control worrying almost every day for 2 weeks or more in a row that you stopped doing some usual activities?*” If participants responded positively to these statements, they were attributed a score of 1, and 0 otherwise. Limitations associated with these outcome variables are addressed later in this article. A descriptive analysis of the sample revealed that 31.7% of students in New Brunswick reported experiencing symptoms of depression in the past 12 months. For Fredericton and the Madawaska regions, these numbers stand at 32.4 and 24.0%, respectively. As for symptoms of anxiety, 33.5% of students in New Brunswick report having felt symptoms of anxiety in the past 12 months, while 34.8% of students in Fredericton and 28.4% of students in the Madawaska region report the same.

### Independent Variables and Preliminary Analyses

The independent variables, also known as determinants, consider the associations found in the above-mentioned literature review, as well as the availability of data from the New Brunswick Student Wellness Survey (NBSWS). They also draw from the NBHC Determinants of Health Model (34), but some variables found in the NBHC model, such as sexual activity and stress, were omitted from the study as they were not documented in the NBSWS. All of these variables are dichotomous, so a value of 1 indicates the presence of the determinant, and a value of 0 indicates the absence of the determinant.

In order to assess the determinants of symptoms of anxiety and depression with logistic regressions, it is recommended to have at least ten times as many participants in the category of the dependent variable with the least number of participants as the total number of variables, or 190 participants (35). With the least frequent modality of the dependent variables having 257 participants, this assumption is met. After simple elimination of cases with at least one missing data item, 68.4% of participants (*N* = 22,329) were included in the analysis. Often, multiple imputation is recommended when a significant proportion of the sample is excluded from the analyses (36). However, power calculations using the G^*^Power software indicate that even with 68.4% of the total sample and a small expected effect size, the statistical power approximates the value of 1. However, it is still necessary to test whether there is an underlying mechanism that could explain the missing data. In other words, it is necessary to test whether the participants included in the analysis have a different rate of symptoms than the participants who were excluded. To do this, a dichotomous variable was created in which participants were given a score of 1 if they were included in the analysis and a score of 0 if they were excluded. This variable was then cross-tabulated with each determinant to assess effect size using phi as an estimator. The largest effect size observed was between gender and depression, where φ = 0.09, which represents a small effect size according to (37). Therefore, it was considered reasonable to assume that the missing data mechanism was random, indicating that the probability of a missing value does not depend on the dependent variable, but could be related to other variables included in the database (35). Since a random distribution of missing data is not problematic, missing data will be handled by simple elimination. Since no participant had a Cook's distance >1, the data set did not contain multivariate outliers. Collinearity was assessed using tolerance values, variance inflation factors (VIFs), and condition indices. The tolerance values were all above the acceptable threshold of 0.10, therefore no collinearity is found (35, 38).

## Results

For both outcome variables, three separate binary logistic regressions were performed. The first regression aimed at identifying the predictive factors at the provincial level, using the entire available sample. The second and third binary logistic regressions were for the two targeted OLMCs. The most striking result from theses analyses stemmed from the Language variable (see Table 2 for complete results). Francophones were 1.42 [1.12, 1.80] times more likely than Anglophones to have anxiety symptoms when in a Francophone minority context, while the difference between the two linguistic groups was statistically insignificant in an Anglophone majority context, with Francophones being 1.12 [0.77, 1.62] as likely to have anxiety symptoms.

**Table 2:** Odds ratios and 95% C.I. for symptoms of anxiety for New Brunswick and two OLMCs.

**Variable – subgroup**	**New Brunswick** ***N* = 22,329**	**Fredericton** **(French OLMC)** ***N* = 4,490**	**Madawaska** **(English OLMC)** ***N* = 1,580**
Francophones	0.97 [0.89, 1.06]	1.42* [1.12, 1.80]	1.12 [0.77, 1.62]
Males	0.31* [0.29, 0.33]	0.30* [0.25, 0.34]	0.37* [0.28, 0.48]
Independent gender	1.03 [0.77, 1.37]	0.79 [0.42, 1.47]	3.55 [0.86, 14.75]
LGBTQ+	1.81* [1.66, 1.98]	1.88* [1.55, 2.28]	2.02* [1.44, 2.84]
Aboriginal status	1.12 [0.98, 1.30]	1.08 [0.83, 1.42]	0.85 [0.43, 1.67]
Immigrant status	0.74* [0.64, 0.86]	0.86 [0.65, 1.14]	2.64* [1.25, 5.60]
Special learning needs	1.72* [1.58, 1.86]	1.67* [1.38, 2.02]	1.70* [1.20, 2.39]
Built and natural environment	0.87* [0.81, 0.93]	0.81* [0.69, 0.94]	0.79 [0.61, 1.26]
Mental fitness needs by family	0.54* [0.50, 0.59]	0.47* [0.40, 0.57]	0.44* [0.32, 0.61]
Mental fitness needs by friends	0.64* [0.59, 0.70]	0.70* [0.58, 0.85]	0.88 [0.62, 1.26]
Mental fitness needs at school	0.56* [0.53, 0.61]	0.50* [0.43, 0.58]	0.61* [0.47, 0.80]
Community safety	0.80* [0.75, 0.85]	0.88 [0.76, 1.03]	1.00 [0.78, 1.28]
Food insecurity	1.69* [1.45, 1.97]	1.67* [1.17, 2.37]	1.86* [1.02, 3.39]
Exercise	0.89* [0.82, 0.97]	0.96 [0.80, 1.15]	0.93 [0.67, 1.29]
Daily or occasional tobacco use	1.15* [1.02, 1.30]	1.33* [1.04, 1.71]	0.68 [0.42, 1.10]
Monthly alcohol use	1.07 [0.98, 1.17]	1.04 [0.85, 1.27]	1.46* [1.09, 1.95]
Monthly cannabis use	1.34* [1.21, 1.49]	1.37* [1.08, 1.72]	1.33 [0.84, 2.08]
Injury Risk	1.28* [1.17, 1.39]	1.26 *[1.04, 1.54]	1.47* [1.11, 1.94]

*Odds ratios followed by an asterisk are significant at the 95% confidence level*.

Other determinants that were significant predictors in one OLMC, but not the other, include the role played by the built and natural environment surrounding the students, and by friends. More specifically, having access to parks and recreational areas, OR = 0.81, 95% CI = [0.69, 0.94], and getting psychological needs, such as autonomy, competence and relatedness, met by their friends, OR = 0.70, 95% CI = [0.58, 0.85], both served as protective factors against anxiety symptoms in the Francophone OLMC, but are statistically insignificant predictors in the Anglophone OLMC. As for individual behaviors, daily or occasional tobacco smoking, OR = 1.33, 95% CI = [1.04, 1.71], and monthly cannabis smoking, OR = 1.37, 95% CI = [1.08, 1.72], both raised the odds of anxiety symptoms in Francophone OLMC, while monthly alcohol consumption did the same in the Anglophone OLMC, OR = 1.46, 95% CI [1.09, 1.95]. Finally, while immigrant status seemed to be a protective factor for anxiety symptoms for the province, OR = 0.74, 95% CI = [0.64, 0.86], this status made youth more vulnerable to anxiety symptoms in the Madawaska region, OR = 2.64, 95% CI = [1.25, 5.60], where Francophones were the majority.

As for the factors associated with symptoms of depression (see Table 3), what stands out the most is, once again, the role played by language. At the provincial level, OR = 0.64, 95% CI = [0.58, 0.70], and in the Anglophone OLMC, OR = 0.54, 95% CI = [0.37, 0.78], Francophones seemed less prone to symptoms of depression than their Anglophone counterparts. However, when looking into the Francophone OLMC, this protective factor was not statistically significant, OR = 0.85, 95% CI = [0.66, 1.10]. In other words, the resistance to symptoms of depression observed in Francophone youth did not occur in a Francophone minority setting. Unfortunately, the observational design of this study does not allow establishing the cause of this decrease in resistance to depression.

**Table 3:** Odds ratios and 95% C.I. for symptoms of depression for New Brunswick and two OLMCs.

**Variable – subgroup**	**New Brunswick** ***N* = 21,697**	**Fredericton** **(French OLMC)** ***N* = 4,348**	**Madawaska** **(English OLMC)** ***N* = 1,562**
Francophones	0.64* [0.58, 0.70]	0.85 [0.66, 1.10]	0.54* [0.37, 0.78]
Males	0.37* [0.35, 0.40]	0.41* [0.35, 0.48]	0.40* [0.30, 0.53]
Independent gender	1.44* [1.04, 2.00]	0.84 [0.43, 1.48]	6.59* [1.19, 36.56]
LGBTQ+	1.83* [1.66, 2.01]	2.02* [1.66, 2.47]	1.77* [1.23, 2.54]
Aboriginal status	1.42* [1.23, 1.64]	1.51* [1.15, 1.98]	1.48 [0.74, 2.95]
Immigrant status	0.89 [0.76, 1.04]	0.89 [0.66, 1.19]	2.77* [1.29, 5.97]
Special learning needs	1.76* [1.62, 1.92]	1.61* [1.32, 1.95]	1.66* [1.15, 2.39]
Built and natural environment	0.89* [0.83, 0.96]	0.89 [0.76, 1.04]	0.93 [0.71, 1.22]
Mental fitness needs by family	0.44* [0.41, 0.48]	0.45* [0.37, 0.53]	0.38* [0.27, 0.53]
Mental fitness needs by friends	0.58* [0.53, 0.63]	0.57* [0.47, 0.69]	0.56* [0.39, 0.80]
Mental fitness needs at school	0.51* [0.47, 0.55]	0.48* [0.41, 0.56]	0.51* [0.38, 0.67]
Community safety	0.72* [0.67, 0.77]	0.78* [0.67, 0.91]	0.92 [0.70, 1.21]
Food insecurity	1.98* [1.68, 2.32]	1.93* [1.32, 2.82]	1.94* [1.04, 3,64]
Exercise	0.87* [0.80, 0.96]	0.87 [0.72, 1.06]	0.91 [0.63, 1.31]
Daily or occasional tobacco use	1.22* [1.08, 1.38]	1.26 [0.98, 1.62]	1.02 [0.61, 1.68]
Monthly alcohol use	1.17* [1.07, 1.29]	1.36* [1.11, 1.68]	1.21 [0.87, 1.66]
Monthly cannabis use	1.70* [1,52, 1.90]	1.70* [1.34, 2.15]	1.63* [1.01, 2.62]
Injury risk	1.34* [1.22, 1.47]	1.30* [1.06, 1.58]	1.44* [1.07, 1.94]

*Odds ratios followed by an asterisk are significant at the 95% confidence level*.

Other determinants that were significant predictors in one OLMC, but not the other, are indigenous status, OR = 1.51, 95% CI = [1.15, 1.98], community safety, OR = 0.78, 95% CI = [0.67, 0.91] and monthly alcohol consumption, OR = 1.36, 95% CI = [1.11, 1.68]. The effect of these determinants is significant in the Francophone OLMC, but not in the Anglophone OLMC. Identifying as indigenous and alcohol consumption both acted as catalysts for symptoms of depression, while feeling like one's community is safe appeared to be a protective factor. In the Anglophone OLMC, being of immigrant status, OR = 2.77, 95% CI = [1.29, 5.97], or considering oneself to be gender-independent, OR = 6.59, 95% CI = [1.19, 36.56], both seemed to increase vulnerability to depression symptoms, while not being the case in the Francophone OLMC. However, due to the small number of students identifying as gender-independent in the Madawaska region (*N* = 23), this result should be interpreted with caution.

## Discussion

The goal of this study was to assess the odds of anxiety and depression symptoms in New-Brunswick's OLMCs by establishing which social determinants of health were important predictive factors of these symptoms. Results revealed that for both anxiety and depression, the linguistic group most vulnerable to symptoms changed depending on which group constituted the social majority. At the provincial level, Francophone and Anglophone students had statistically equal odds of experiencing symptoms of anxiety, but in the Francophone OLMC, Francophones found themselves to be 1.42 [1.12, 1.80] times more vulnerable to these symptoms. Possible explanations for this association include assimilation pressures due to the importance of English in an Anglophone environment (24, 25), insecurity linked to the loss of Francophone or Acadian identity (39) or even collective angst, “a group-based emotion that stems from concern for the future vitality of one's social group” (40). When Francophone students were in a Francophone context, as is the case in the Madawaska region, the increased vulnerability to anxiety symptoms found in minority Francophones was muted. This result is consistent with theories brought forward by (26), according to which the vulnerabilities generally associated with minority groups are overshadowed by the benefits of being the majority group at the local level.

As for symptoms of depression, Francophone students seemed less vulnerable than their Anglophone counterparts in general. In fact, Francophone students were only 0.64 [0.58, 0.70] times as likely as their Anglophone counterparts to experience symptoms of depression. However, when placed in a Francophone OLMC, this difference vanished, raising the hypothesis of assimilation or cultural identity loss once more. Interestingly, symptoms of anxiety and depression followed the same tendency, as Francophone students in the OLMC had significantly higher odds of experiencing symptoms than the Anglophone students, but in the Francophone majority setting, both linguistic groups had statistically similar odds. Finally, students who reported being immigrants seemed considerably more vulnerable to symptoms of anxiety and depression in the Madawaska region than in the Fredericton region. No literature pertaining to differences in welcoming immigrants between the Francophone and Anglophone population could be found, but recent immigration trends could offer a plausible explanation. With New Brunswick's recent uptake in Chinese and Syrian refugees, most of these newcomers settle in larger cities, such as Fredericton, Moncton or Saint John (41). In fact, between 2011 and 2016, Fredericton welcomed 2,625 newcomers, while Edmundston, the largest city in the Madawaska region, welcomed only 200. In total, 8,255 immigrants live in Fredericton, which represents 8.2% of its population, while only 875 immigrants live in Edmundston, representing 3.9% of its population (41). The difference in the number of immigrants between the two regions could explain why immigrants in the Fredericton area fared much better than the immigrants in the Madawaska region, as social psychology theories tend to agree that larger ingroups increase group identification and, in turn, the well-being of its members (42). Income-based inequities in access to mental health services may also be a root cause (43). Looking beyond the OLMCs, some general tendencies emerged for all New Brunswick youth, regardless of social group status. First and foremost, mental fitness, which refers to the personal sense of psychological wellness provided by friends, family and the school environment in relation to the psychological needs of autonomy, competence and relatedness, appears to be an important protective factor against anxiety and depression symptoms. Except for mental fitness provided by friends in the Madawaska region, all three types of mental fitness seemed to reduce the odds of having symptoms for both anxiety and depression, with odd ratios ranging from 0.38 to 0.70. Moreover, having psychological needs met by family, friends and/or by school staff was a significant protective factor for students across the province.

Gender was a strong predictive factor of symptoms, with fewer boys reporting symptoms for both anxiety and depression. However, previous studies suggest that differences may not lie in the prevalence of mental health problems, but rather in the stigma attached to admitting to having mental health issues (44, 45). Differences in anxiety and depression symptoms were also found between heterosexual youth and youth from the LGBTQ+ community. At the provincial level, LGBTQ+ community members were 1.81 [1.66, 1.98] and 1.83 [1.66, 2.01] times more likely to report anxiety and depression symptoms, respectively, than their heterosexual peers. These results are consistent with previous literature on the subject (10, 46).

Food insecurity, characterized here by kids going to school or bed hungry due to a lack of food in the house, plays an important role in predicting symptoms of anxiety and depression at the provincial level, with youth with food insecurity having 1.69 [1.45, 1.97] times the odds of having symptoms of anxiety and 1.98 [1.68, 2.32] the odds of reporting depression symptoms than those without. Unfortunately, food insecurity is quite common in New Brunswick, with one in five children experiencing it in 2014 (5).

Finally, since the collection of this data, cannabis has become legal in Canada for adults over the age of 19, except for Alberta and Quebec, where the legal age is 18. Although the rules and regulations around cannabis use for minors have remained unchanged, one can assume that the availability and the diminishing stigma attached to cannabis use in the country might impact cannabis use in minors. Regardless, in this study, cannabis use was associated with 1.34 [1.21, 1.49] times the odds of having anxiety symptoms and 1.70 [1,52, 1.90] times the odds of having depression symptoms.

### Limitations

Survey-based studies are limited by their observational design, providing weak evidence for causal relationships. On the flip side, surveys with such an important number of participants reduce the odds of having a non-representative sample of the population. A second limitation to this study comes from the dichotomous nature of the variables. Although this simplifies the interpretation of the logistic regressions, there is inevitably a loss of information when variables are of dichotomous nature. Similarly, the chosen outcome variables were limited in their ability to identify students who were truly experiencing anxiety and depression, as their description of the symptoms were not exhaustive, and their binary nature eliminated nuances between those who experienced many symptoms and those who only experienced a few. Unfortunately, little could be done to counter these limitations, as we had no role in the conception of the survey. Future studies should rely not only on the available data but combine these results to prior knowledge on the predictive values of these social determinants on mental health.

## Conclusion

Regardless of the linguistic context in which children become young adults, some social determinants seem better than others at predicting symptoms of anxiety and depression. For example, having basic psychological needs such as autonomy, competence and relatedness met by either family, friends or at school, is a strong protective factor against negative mental health symptoms. On the other hand, non-heterosexual orientation or food insecurity can lead to increased odds of reporting anxiety or depression symptoms. These social determinants of health are important to consider in any ethnic or linguistic context. This study's main contribution, however, is its ability to identify factors associated with symptoms of anxiety and depression in OLMCs, and therefore providing guidelines for actions aimed at reducing mental health disparities in specific regions and for specific student profiles. Examples of this are the protective role of the built and natural environment surrounding students in the Francophone OLMCs or the negative effects of being an immigrant and youth alcohol consumption in Anglophone OLMCs.

## Data Availability Statement

The data analyzed in this study is subject to the following licenses/restrictions: Permission to access the data from the NBHC is needed. Requests to access these datasets should be directed to NBHC, info@nbhc.

## Ethics Statement

Ethical review and approval was not required for the study on human participants in accordance with the local legislation and institutional requirements. Written informed consent to participate in this study was provided by the participants' legal guardian/next of kin to the New Brunswick Health Council.

## Author Contributions

JBD worked on all parts of the study and wrote the article. JB helped plan the study and shape the theoretical context. S-EE oversaw the preliminary and main analyses. All authors contributed to the article and approved the submitted version.

## Conflict of Interest

The authors declare that the research was conducted in the absence of any commercial or financial relationships that could be construed as a potential conflict of interest.

## Abbreviations

OLMC: official language minority community
NBHC: new brunswick health council
NBSWS: new brunswick student wellness survey
SES: socioeconomic status
OR: odds ratio
95% CI: 95% confidence interval.
